# Cellular mechanisms mediating the anti-cancer effects of carnosol on gingiva carcinoma

**DOI:** 10.1038/s41598-024-60797-x

**Published:** 2024-05-28

**Authors:** Nassima Gassib, Hawraa Issa, Lionel Loubaki, Sarah Behaz, Mikhlid H. Almutairi, Mahmoud Rouabhia, Abdelhabib Semlali

**Affiliations:** 1 Groupe de recherche en écologie buccale, Faculté de médecine dentaire, Université Laval, Québec, QC G1V 0A6 Canada; 2 Héma-Québec, 1070, Avenue des Sciences-de-la-Vie, Québec, QC G1V 5C3 Canada; 3 Zoology Department, College of Science, King Saud University, P.O. Box 2455, 11451 Riyadh, Saudi Arabia

**Keywords:** Carnosol, Oral cancer, Gingiva carcinoma, Cell cycle, A poptosis, Oxidative stress, Migration, Signaling pathways, Cancer, Cell biology, Molecular biology, Plant sciences

## Abstract

Carnosol, a rosemary polyphenol, displays anticancer properties and is suggested as a safer alternative to conventional surgery, radiotherapy, and chemotherapy. Given that its effects on gingiva carcinoma have not yet been investigated, the aim of this study was to explore its anti-tumor selectivity and to unravel its underlying mechanisms of action. Hence, oral tongue and gingiva carcinoma cell lines exposed to carnosol were analyzed to estimate cytotoxicity, cell viability, cell proliferation, and colony formation potential as compared with those of normal cells. Key cell cycle and apoptotic markers were also measured. Finally, cell migration, oxidative stress, and crucial cell signaling pathways were assessed. Selective anti-gingiva carcinoma activity was disclosed. Overall, carnosol mediated colony formation and proliferation suppression in addition to cytotoxicity induction. Cell cycle arrest was highlighted by the disruption of the c-myc oncogene/p53 tumor suppressor balance. Carnosol also increased apoptosis, oxidative stress, and antioxidant activity. On a larger scale, the alteration of cell cycle and apoptotic profiles was also demonstrated by QPCR array. This was most likely achieved by controlling the STAT5, ERK1/2, p38, and NF-ĸB signaling pathways. Lastly, carnosol reduced inflammation and invasion ability by modulating IL-6 and MMP9/TIMP-1 axes. This study establishes a robust foundation, urging extensive inquiry both *in vivo* and in clinical settings, to substantiate the efficacy of carnosol in managing gingiva carcinoma.

## Introduction

Oral cancer (OC) was ranked as the 16th most common cancer in the world, with 34,864 new global cases in 2018. This number increased drastically to 377,713 in 2020, thus highlighting the rapidly increasing prevalence of oral tumors^1–3^. Strong disparities in the incidence rates of OC between world regions have been unraveled. Melanesia and South-Central Asia exhibited the highest incidence of OC, accounting for approximately 52% of all global cases, followed by Eastern Europe, Australia, and New Zealand^3^. Among head and neck cancers, oral squamous cell carcinoma (OSCC) arising in the squamous cells of the epithelial surface is the most common, accounting for 90% of all OCs, with an estimated 5 year overall survival rate of approximately 50% of patients^4^. Tobacco exposure, betel nut use, alcohol use, and human papillomavirus (HPV) infections are believed to be the main factors of OSCC carcinogenesis^5–8^. Family predisposition is another factor to be taken into consideration.

Indeed, often diagnosed at a late stage, the prognosis of these oral tumors remains poor, even in leading countries, despite therapeutic progress and increased prevention efforts^9^. Treatment of OSCC during its early stages showed the most favorable prognosis, with an estimated 3 year patient survival rate of 92%^10^. The currently used treatment plans recommend squamous cell carcinoma removal from the oral cavity and neck, primarily through surgery^11^. This allows a detailed examination of the tissue for histopathological characteristics such as depth of invasion. Further investigation into the spread to lymph nodes might call for radiation use or chemotherapy^11^. Radiotherapy is frequently used to treat small or large oral tumors in the event of metastases, pathological limits, and certain inoperable cases due to large tumor sizes or inaccessibility by surgery^12^.

On the other hand, chemotherapy is most often found in a therapeutic panel of advanced stages. Although chemotherapy is often associated with better overall survival rates in patients with OC, its clinical use is limited by its severe and toxic dose-dependent side effects. These affect the patient's quality of life and might require lowering the drug dosage or even stopping its administration in certain situations, therefore weakening treatment outcomes^13,14^. OC patients are also known to develop a strong tendency toward drug resistance and metastasis^15^. Owing to the shortage of effective treatments that are free of side effects, finding new anticancer drugs for the treatment of OC is considered an emergency approach.

Plant-derived compounds, also known as phytochemicals, which were largely used by ancient civilizations, are currently emerging as safe treatment alternatives owing to their low cost, proven efficacy and reduced adverse effects^16^. The number of articles published on phytotherapy has skyrocketed on PubMed over the past two decades, as multiple molecules have been extensively studied in diverse fields. Carnosol, a naturally occurring phytopolyphenol, was first isolated from sage (Salvia carnosa) in 1942, but its chemical structure was later established in 1964^17^. In addition to carnosol, rosemary and sage are known to contain other active compounds, including carnosic acid, rosmanol, and rosmarinic acid^18^. Carnosol was reported to reduce complications linked to multiple pathological conditions, including inflammation^19^, microbial infection^20,21^, and cancer development^22^. Many recent studies have investigated the potential preventive and therapeutic roles of carnosol against various cancers, including prostate, colon and breast cancers^23–26^. During carcinogenesis, carnosol not only limits colony growth but also induces cytotoxicity, cell cycle arrest^23–25^, oxidative stress, autophagy, and apoptosis by controlling multiple signaling pathways and transcription factors^23–26^.

Even though recent efforts reported potential cytotoxic effects on the head and neck squamous cell carcinoma lines Hep-2 and SCC-15 and provided important insights on the implication of FOXD1-mTOR as well as the COX-2 signaling pathways^27,28^, no research has investigated in depth the effect of carnosol on gingiva carcinoma development nor evaluated the molecular mechanisms mediating its effects. Therefore, the aim of this study was to confirm effectiveness of carnosol on gingiva carcinoma *in vitro* and to reveal its impact on multiple tumorigenesis mechanisms, particularly cell proliferation, cell cycle, apoptosis, oxidative stress and migration. In addition, key signaling pathways linked with oncogenesis were also analyzed.

## Materials and methods

### Cell culture

The Ca9-22 human gingival epithelial carcinoma cell line was purchased from the RIKEN BioResource Research Center (Tsukuba, Japan). The cells were cultured in RPMI 1640 medium (Corning, 10-013-CV) supplemented with 5% fetal bovine serum (FBS), 0.2% penicillin-streptomycin (P/S), and 0.2% fungizone (F). Tongue cancer cells (SCC-9) collected from ATCC (ATCC, CRL1629) were also used in this study. A non-oncogenic human epithelial cell line, GMSM-K, was provided by Dr. Daniel Grenier (Laval University, Québec City, Canada). SCC-9 and GMSM-K cells were grown in DMEM/F-12 medium supplemented with 10% FBS, 0.2% P/S, and 0.2% F. All cell lines were exposed to increasing concentrations of the carnosol compound (MCE, HY-N0643) for 24 h. Cells were also incubated with the N-acetylcysteine agent, a ROS scavenger, also known as NAC (A7250, Millipore Sigma Canada), at 10 mM concentration.

### MTT cell viability and proliferation assay

After 24 h of carnosol treatment, the MTT solution (M-2128, Sigma) was added at 0.5 mg/ml for 3 h at 37 °C in the dark^29,30^. Afterward, the formed formazan crystals produced by metabolically active cells were dissolved using 1 ml of isopropanol, and then the optic density was measured at 550 nm. Viable proliferating cell levels were determined using the following formula: % of cell viability = [(OD550 nm (treated cells)−OD (Blank))/(OD (control cell)−OD (Blank))] × 100.

### Hoechst nuclear staining

As described by Semlali et al.^31,32^ and Contant et al.^33^, the cells were washed with phosphate-buffered saline (PBS) and then fixed for 2 min with a solution consisting of 75% methanol and 25% acetic acid. The cells were rewashed before being refixed with the same solution for 5 min. Three additional wash cycles were performed prior to incubation with the Hoechst nuclear stain (33342, Thermo Fisher Scientific) for 15 min. Finally, the excess product was eliminated after adding PBS, and the cells were observed under a fluorescent microscope.

### LDH cytotoxicity assay

The LDH (Lactate Dehydrogenase) cytotoxicity detection kit (11644793001, Roche) was used in accordance with the manufacturer’s specifications. As described by our previous studies^32,34^, 50 μL of culture supernatants was transferred in triplicate to a 96-well plate and then supplemented with an equal volume of reconstituted substrate mix. The latter consisted of a dye solution and catalyst. The plates were then incubated for 30 min at room temperature in the dark, resulting in the release of LDH enzyme from damaged cells that enabled the formation of a red formazan product that was easily detected at 490 nm. The LDH activity was calculated using the following formula: % of LDH activity = [treated cells (absorbance) −negative control (absorbance)]/[positive control (absorbance) −negative control (absorbance)] × 100.

### Colony formation assay

A total of 2 × 10^3^ cells were exposed to different carnosol concentrations for 14 days before being fixed with 100% cold ethanol for 10 min at 4 °C. Afterward, the cells were washed twice with cold PBS and then stained with 0.5% crystal violet (548-62-9, Sigma) for 30 min. The plates were subsequently washed with deionized water, left to dry at room temperature, and photographed.

### Cell morphology

As described by Loubaki et al.^29^, 4 × 10^3^ OC cells (Ca9-22) were seeded on sterile glass coverslips and exposed to 0, 20, and 50 μM of Carnosol for 24 h. The slides were then photographed using a digital camera (COOLPIX 995, Nikon).

### Annexin V-propidium iodide apoptosis assay

Apoptosis was measured using the annexin V-propidium iodide kit (640932, BioLegend). Briefly, the cells were incubated with 5 μL annexin V-APC and/or 5 μL propidium iodide for 15 min at room temperature before resuspension using 400 μL of the annexin V binding buffer. Flow cytometry analysis using either BD LSR II or BD FACSCanto II cytometer equipped with FACSDiva software v. 6.1.3. ensured the sorting of the collected events as a percentage of viable and dying cells at the early apoptotic, late apoptotic, and necrotic stages^29–34^.

### Caspase activity assay by flow cytometry

To investigate the mechanism by which carnosol induced OC apoptosis, caspase activity was evaluated using a caspase detection kit (QIA90, Calbiochem), in accordance with the manufacturer's instructions. As described in our previous study^34^, Ca9-22 cells were trypsinized and washed twice with PBS after supplementation with carnosol for 24 h. The cells were then incubated in the presence of 1 μL of FITC-VAD-FMK for 1 h at 37 °C in a 5% CO_2_ incubator. After three washing cycles with PBS, the cells were analyzed using flow cytometry.

### Real-time PCR

RNeasy mini kit (74104, Qiagen) was used to extract total RNA from the cells. A total of 2 μg RNA was reverse-transcribed into cDNA copies with the iScript Reverse Transcription Supermix for real-time qPCR (1708841, Bio-Rad). The RNA concentration and purity were determined using a Nanodrop 8000 spectrophotometer (Thermo Fisher Scientific). The following primers (Table 1) and the IQ SYBER Green Supermix (64204590, Bio-Rad) were used to ensure the quantification of the corresponding RNA levels. Transcript levels were analyzed using Bio-Rad CFX Manager 3.1 and normalized to GAPDH. PCR analysis was performed using the 2-∆∆CT method for relative gene expressions.

**Table1.** Descriptions of the primer sequences used for the qRT-PCR reactions.

Gene	Primer sequence (5’–3’)	Amplicon size (bp)	Tm (°C)
IL-6	Fw: 5'-TCTCCACAAGCGCCTTCG-3’ Rv: 5'-CTCAGGGCTGAGATGCCG-3’	203	60
MMP-9	Fw: 5'-ATTTCTGCAGCTCTGTGTGAA-3’Rv: 5'-TGAATTCTCAGCCCTCTTCAA-3’	263	60
TIMP-1	Fw:5′-ATCCTGTTGTTGCTGTGGCTGATAG-3′Rv:5′-TGCTGGGTTAACTCTTTATTTCA3′	689	58
GAPDH	Fw:5'GGTATCGTCGAAGGACTCATGAC-3’Rv:5'-ATGCCATGAGCTTCCCGTTCAGC-3’	180	60

### Cell cycle and apoptotic gene expressions using the RT2 profiler PCR arrays

RT2 Profiler PCR arrays (PAHS-020ZD-6 and PAHS-012ZD-6, Qiagen) were used to screen for a comprehensive panel of relevant genes involved in cell cycle and apoptosis, as previously described by Loubaki et al.^29^ and Tazi et al.^35^. Briefly, 3 × 10^5^ OC cells were stimulated with 10 µM carnosol (IC_50_ representative value) for 24 h. PCR was performed using PCR mixture components, in accordance with the manufacturer’s instructions. Finally, CT values were analyzed using the Qiagen software (http://www.qiagen.com/geneglobe). Relative gene expression data analysis was performed using the 2^−∆∆CT^ method, and the fold change between the control and carnosol-treated cells was evaluated. Only genes with a fold regulation > 2 compared with the controls were considered positively or negatively regulated. Gene expression values were normalized according to the automatic selection of a full panel of reference genes.

### Total ROS activity assay

Oxidative stress was assessed by flow cytometry using an intracellular total ROS (Reactive oxygen species) activity assay kit (9144, Immunochemistry Technologies). After stimulation with carnosol, the cells were collected through the action of trypsin and then resuspended in 490 μL of medium supplemented with 10 μL of total ROS stain solution. Ca9-22 cells were then incubated in the dark for 1 h at 37 °C. The green ROS dye excitation and emission wavelengths were 490 and 520 nm, respectively^30,32^.

### Measurement of mitochondrial superoxide

Mitochondria-mediated ROS production was assessed using the MitoSOX-Red Mitochondrial Superoxide Indicator (M36008, Invitrogen). Ca9-22 cells were harvested and incubated with 5 μM of the mitochondrial dye for 30 min at 37 °C. After centrifugation, the pellet was resuspended in 1 ml of PBS for flow cytometry analysis. “LSRII” and “CantoII” cytometers (BD Bioscience) equipped with FACS Diva software v. 6.1.3 were used to calculate the percentage of MitoSox-positive cells^30,32^.

### Intracellular glutathione evaluation

The intracellular GSH assay kit (9137, Immunochemistry Technologies) was used for this purpose. The Ca9-22 cell suspension was incubated with 1:200 ThioBright Green reagent at 37 °C for 30 min^33^. After being centrifuged and resuspended in PBS, the cells were analyzed with a BD FACSCalibur flow cytometer (BD Bioscience).

### Western blotting

The effects of carnosol on various proteins involved in cell cycle and apoptosis were evaluated using Western blotting analysis. Briefly, cells were harvested, the proteins were extracted using the RIPA lysis buffer, and protein concentration was determined using the Bradford assay (5000006, Bio-Rad). Cell samples were migrated through 8–15% acrylamide gels. After electrophoretic separation, proteins were transferred to nitrocellulose membranes and blocked in a 5% milk solution for 1 h. The membranes were then placed overnight at 4 °C with the appropriate concentrations of antibodies. β-Actin (A5441, Sigma), GAPDH (Sc-47724), p53 (sc-263), Bcl-2 (sc-509), Bax (sc-7480), total PARP-1 (sc-8007), cleaved caspase-3 (9664S, cell signaling), c-myc primary antibodies (51-1485GR, BD Pharmingen) were used for this study. Goat anti-mouse (554,002) and anti-rabbit secondary antibodies (554,021) were bought from BD Pharmingen (Mississauga, ON, Canada). Band detection was enabled with the Clarity Western ECL Substrate (1705061, Bio-Rad) using a VersaDoc Imaging System (Bio-Rad).

### Scratch assay

The carnosol effect on cell migration was investigated after the application of a scratch in a cell monolayer, as described in our previous works^31,36^. The wound diameter was measured over 6 h to evaluate healing properties using the ImageJ 1.47v software, and images were taken using an optical microscope to examine the effects of the drug.

### Zymography

Nonreducing sample buffer was added to cell supernatants before migration in a polyacrylamide gel containing 1% gelatin (424865, J.T. Baker). After two washing cycles, the gels were incubated overnight with the developing buffer at 37 °C. Coomassie Brilliant Blue R-250 staining solution (161–0436, Bio-Rad) was then supplemented for 30 min at room temperature to reveal gelatin degradation by active gelatinase enzymes. The destaining buffer allowed the detection of inactive pro-MMP9 bands.

### Study of cancer signaling pathways

Flow cytometry was used to analyze the phosphorylation levels of NF-κB, STAT5, p38, and ERK in Ca9-22, as described in our earlier studies^29,34^. After treatment with carnosol, cells were washed with PBS + 2% FBS and fixed with 1.5% formaldehyde. They were permeabilized by incubation with methanol/PBS (90% v/v) on ice for 20 min before labeling for 30 min with 5 µL of Alexa Fluor 647 mouse anti-human pSTAT5 (clone 47/Stat5[pY694]), pERK1/2 (clone 20A), pp38 (clone 36/389pT180/pY182) purchased from BD Biosciences, and pNF-κB-p65 (Clone B33B4WP, Thermo Fisher Scientific). After washing three times, the cells were suspended in 300 µL of PBS before flow cytometry analysis (BD Accuri C6, BD Biosciences).

### Statistical analysis

All experiments were performed more than three times. The significant differences between the experimental (treated with carnosol) and control groups (untreated) were evaluated using the student t test in the GraphPad Prism 7 software. Data are represented as the mean of the percentage ± SEM. *p < 0.05 is considered statistically significant. For the PCR and RT2 Profiler PCR array analyses, the relative gene expression data analysis was performed using the 2^−∆∆CT^ method.

## Results

### Selective effects of carnosol on gingiva carcinoma

**Figure 1:**
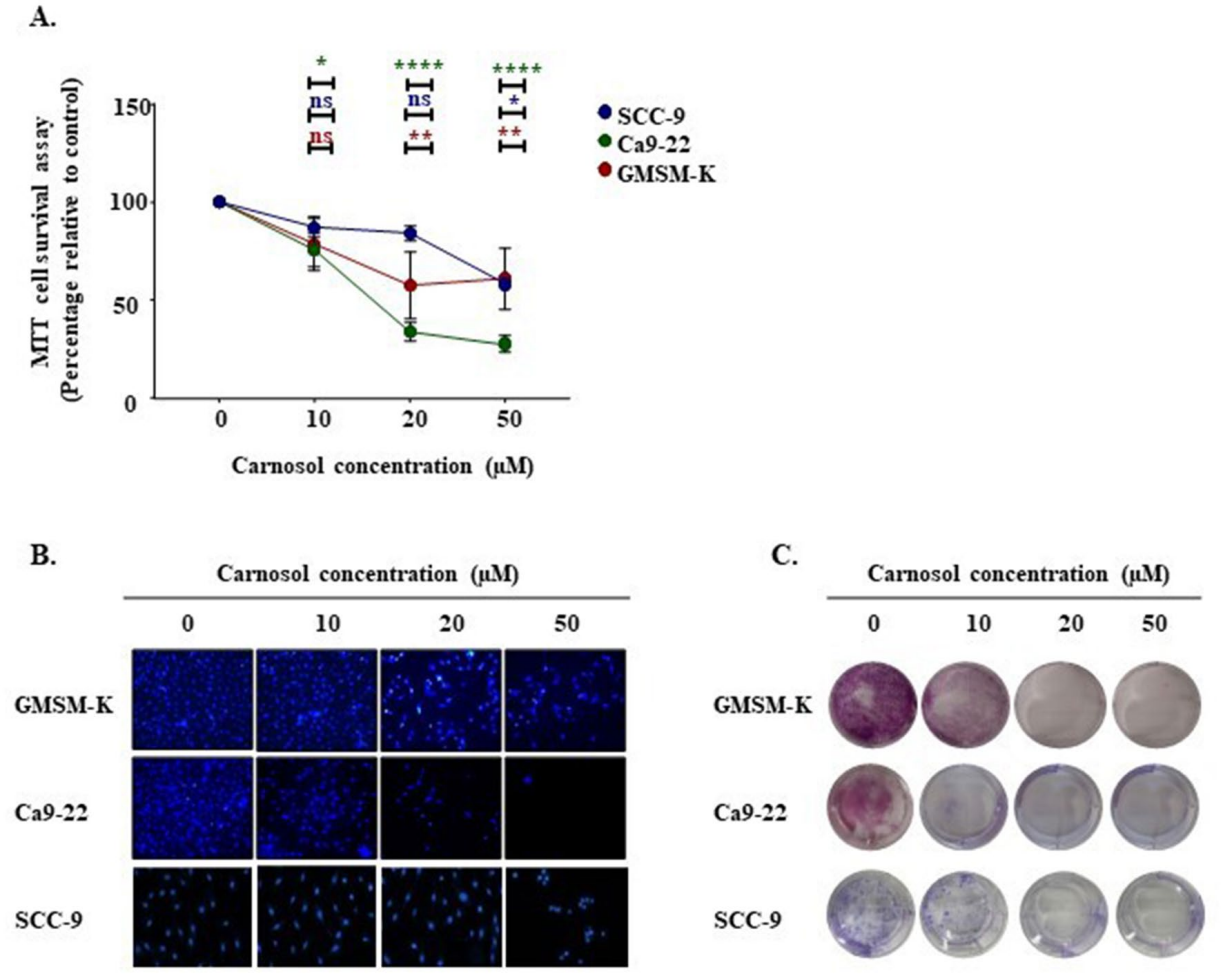
Effect of carnosol on cell viability, proliferation, and colony formation. (**A**) MTT cell proliferation and viability assay. The presented data are expressed as mean ± SEM values from 4 to 14 independent experiments. *p < 0.05, **p < 0.01, ***p < 0.001, and ****p < 0.0001 are considered statistically significant. (**B**) Hoechst nuclear staining performed to measure cell growth after 24 h treatment of carcinoma and normal cell lines with different carnosol concentrations (n = 3). (**C**) Crystal violet assay for evaluating colony formation capacity upon supplementation with carnosol for 14 days (n = 3).

To evaluate the effects of carnosol on human gingival epithelial carcinoma cells (Ca9-22), tongue carcinoma (SCC-9), and human epithelial cells (GMSM-K) proliferation, MTT assay was performed. Treating cells with different concentrations of carnosol (0, 10, 20, and 50 µM) for 24 h unveiled cancer-selective effects against gingiva carcinoma. Moreover, carnosol inhibited Ca9-22 proliferation in a dose-dependent manner. The values decreased from 100 to 90.2% ± 7.8%, 78.86% ± 11.4%, 35.68% ± 13.17%, 25.92% ± 11.34%, and 10.3% ± 5.82%. Carnosol showed a 50% inhibition of Ca9-22 viability at a concentration of approximately 17 µM, making it the half-maximal inhibitory concentration (IC50). Indeed, even at the highest concentration, GMSM-K and SCC-9 still displayed good resistance to this polyphenol (Fig. 1A). Nuclear staining at the same time points also highlighted the SCC-9 resistance and tumor specificity of carnosol to gingiva carcinoma. Treatment with 10 µM carnosol led to a significant decrease in the number of cancer cells, whereas a less intense effect was observed in GMSM-K and SCC-9 cells (Fig. 1B). Furthermore, 14 days of exposure to carnosol eliminated colony formation potential at high concentrations (20 and 50 μM), and 10 μM carnosol was found to be highly efficient against Ca9-22 cells (Fig. 1C).

### Carnosol exhibits cytotoxic effects and modulates Ca9-22 cell cycle proteins

**Figure 2:**
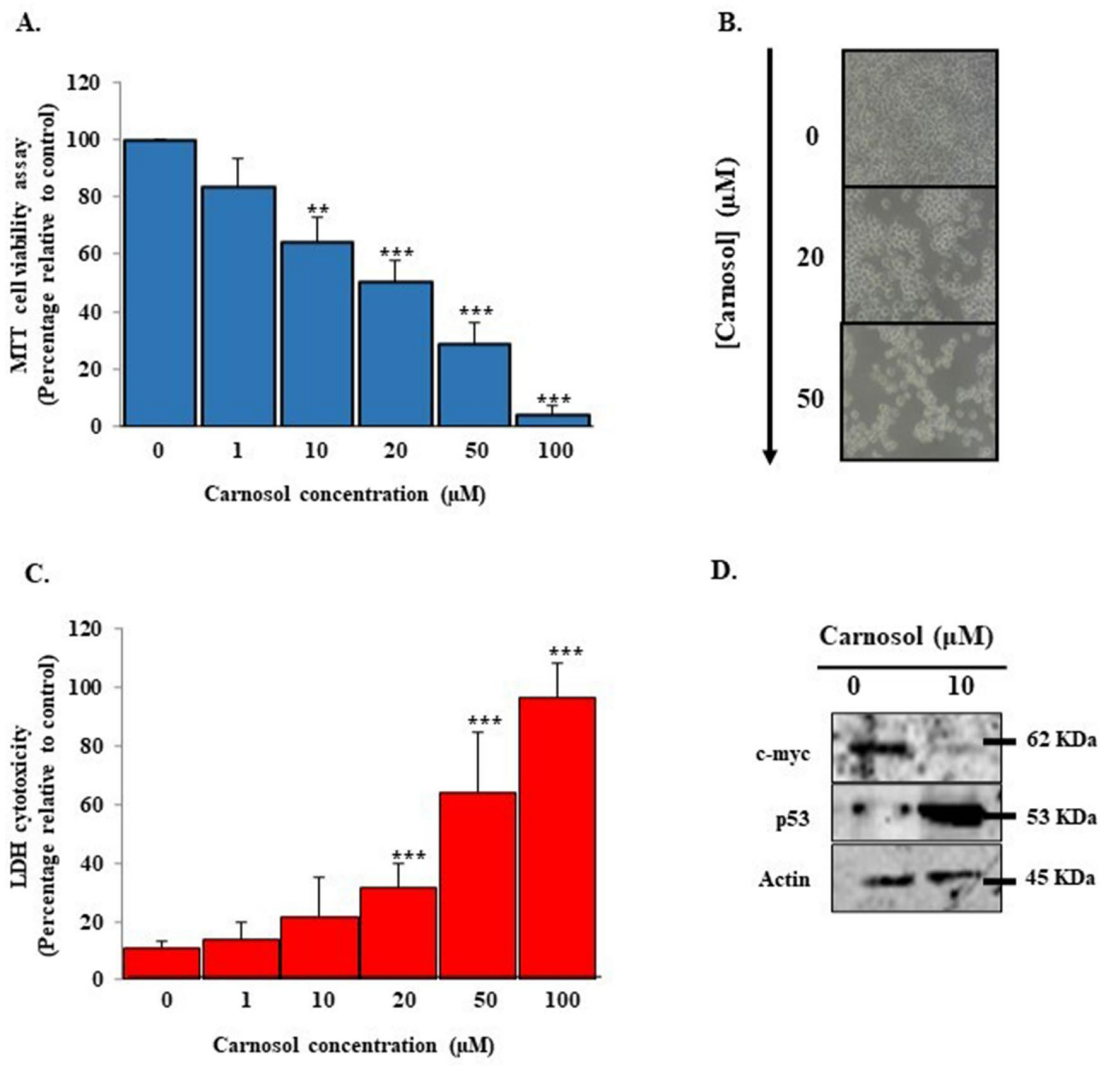
Cytotoxicity evaluation and effect of carnosol on Ca9-22 cell cycle markers. (**A**) MTT viability and proliferation assay results (n = 6) correlated with (**B**) Ca9-22 cell morphology results after the addition of carnosol (n = 4). (**C**) LDH enzyme activity assessment expecting direct cytotoxic effects (n = 10). (**D**) Protein expression levels of p53, actin, and c-myc measured using western blotting analysis (n = 3). The presented data are mean ± SEM values from 3 to 10 independent experiments. *p < 0.05, **p < 0.01, ***p < 0.001, and ****p < 0.0001 are considered statistically significant.

The effects of carnosol on Ca9-22 viability and cell proliferation are linked to cell death induction and possible cell cycle arrest (Fig. 2A). Our results show that carnosol causes morphological changes in Ca9-22 cells and renders them smaller in size with visible damage in the nucleus, which suggest DNA damage in gingiva carcinoma cells (Fig. 2B). In this context, the lactate dehydrogenase (LDH) assay demonstrated dose-dependent cytotoxicity upon treatment with 20, 50, and 100 µM carnosol. The percentage increased from 22.1% ± 1% to 59% ± 6.3%. The IC50 was also approximately 17 µM (Fig. 2C). On the other hand, carnosol disrupted the expressions of the c-myc and p53 genes. More specifically, activating the p53 tumor suppressor and inhibiting the c-myc oncogene are most likely responsible for cell cycle arrest (Fig. 2D).

**Figure 3:**
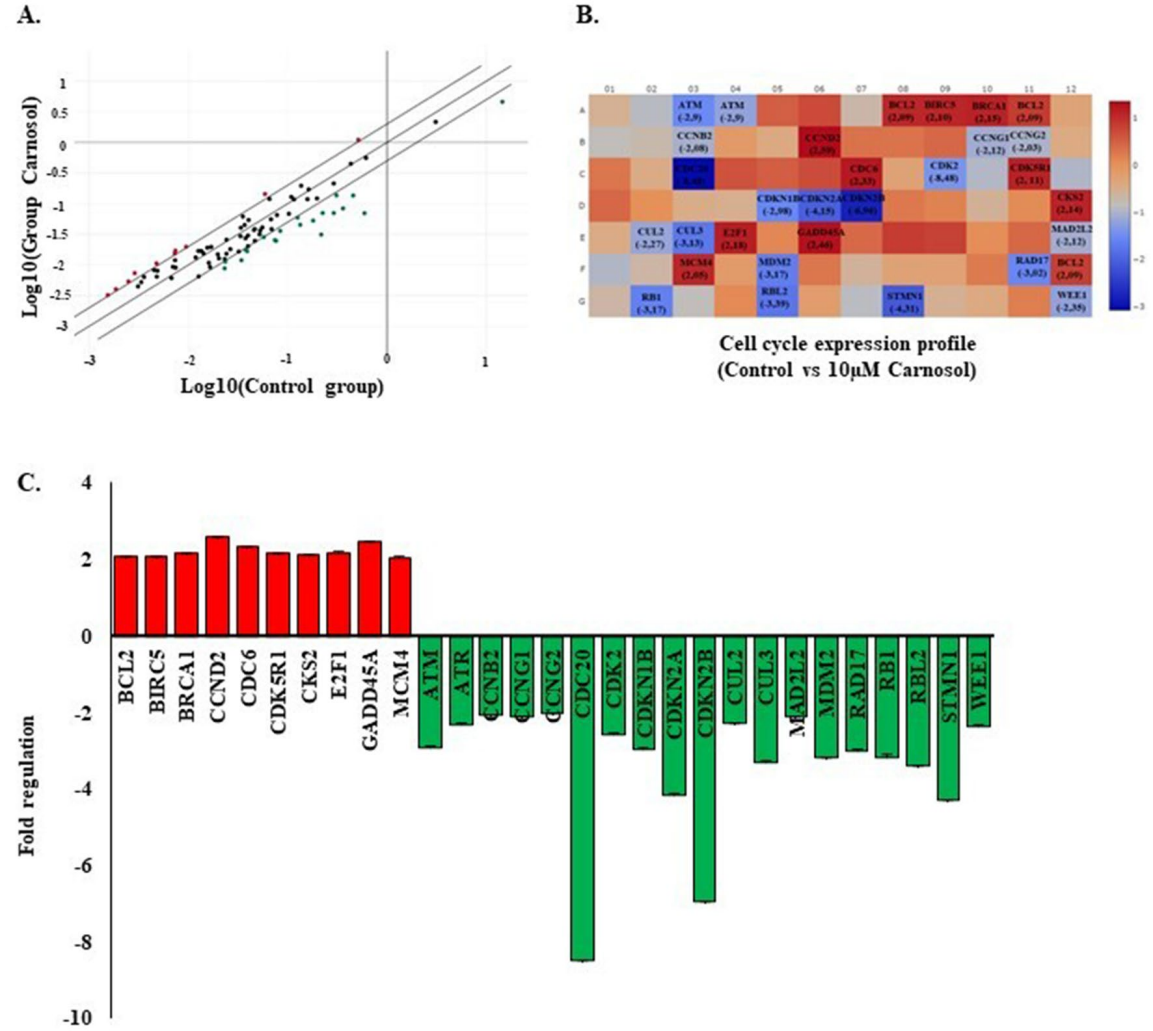
Effect of carnosol on Ca9-22 cell cycle marker expression profile. (**A**) QPCR array screening for 84 cell cycle genes after incubation with 10 μM carnosol. (**B**) and (**C**) Summary of positively and negatively modulated genes. Only fold regulation values > 2 were considered. The presented data are from two experiments.

A comprehensive study estimating carnosol effect on cell cycle genes expression was performed using RT2 Profiler cell cycle array (Qiagen). This allowed for the analysis of 84 genes involved in different cell cycle phases. The data revealed 29 modified genes (Fig. 3A,B). The classification of these markers according to their role has shown that four genes are implicated in the G1 phase and G1/S transition, including cyclin dependant kinase inhibitor 1B (CDKN1B/P27KIP1*)* (−2.98-fold), Cullin 2 (CUL2*)* (− 2.27-fold), Cullin 3 (CUL3*)* (− 3.31-fold), and E2F Transcription Factor 1 (E2F1*)* (2.18-fold). Three genes are involved in the S phase and DNA replication: Cell division cycle 6 (CDC6*)* (2.33-fold), Minichromosome Maintenance Complex Component 4 (MCM4) (2.05-fold), and WEE1 (− 2.35-fold). Two genes are involved in the G2 phase and G2/M transition: Baculoviral IAP Repeat Containing 5 (BIRC5/Survivin) (2.10-fold) and cyclin dependent Kinase 5 regulatory subunit 1(CDK5R1) (2.16-fold). Four markers are involved in the M phase, including the Cyclin B2 (CCNB2) gene (− 2.08-fold), cell division cycle 20 (CDC20) (− 8.48-fold), cell division cycle 6 (CDC6) (2.33-fold), and Stathmin 1 (STMN gene (− 4.31-fold). Of the 29 selected genes, 15 are specifically involved in cell cycle checkpoint and cell cycle arrest: ATM (Ataxia-Telangiesctasia Mutated) (− 2.9-fold), ATR (Ataxia telangiectasia and Rad3-related)(− 2.31-fold), BRCA1 (Breast cancer1) (2.15-fold), CCNG2 (Cyclin G2)(− 2.05-fold), CDKN1B/p27KIP1 (− 2.98-fold), CDKN2A/p16INK4A (− 4.15-fold), CDKN2B/p15INK4b (−6.96-fold), CUL2 (− 2.27-fold), CUL3 (−3.31-fold), GADD45A(growth arrest and DNA damage-inducible 45 alpha) (2.46-fold), MAD2L2 (Mitotic Arrest Deficient 2 Like 2) (− 2.12-fold), MDM2 (Murine Double Minute 2) (− 3.18-fold), RAD17 (− 3.02-fold), RB1 (− 3.17-fold), WEE1 (− 2.35-fold). Finally, 12 genes are known as key cell cycle regulators: ATR (− 2.31-fold), BCL2, CCNB2, CDC20 (− 8.48-fold), CDC6 (2.33-fold), CDK2 (− 2.57-fold), CDK5R1 (2.16-fold), CDKN1B/p27KIP1 (− 2.98-fold), E2F1 (2.18-fold), GADD45A (2.46-fold), RB1 (− 3.17-fold), and WEE1 (− 2.35-fold). Meanwhile, four genes are known as negative modulators: ATM (− 2.9-fold), BRCA1 (2.15-fold), CDKN2B (− 6.96-fold), and RBL2 (− 3.39-fold) (Fig. 3C).

### Carnosol induces Ca9-22 cell death via apoptosis by triggering the caspase signaling pathway

**Figure 4:**
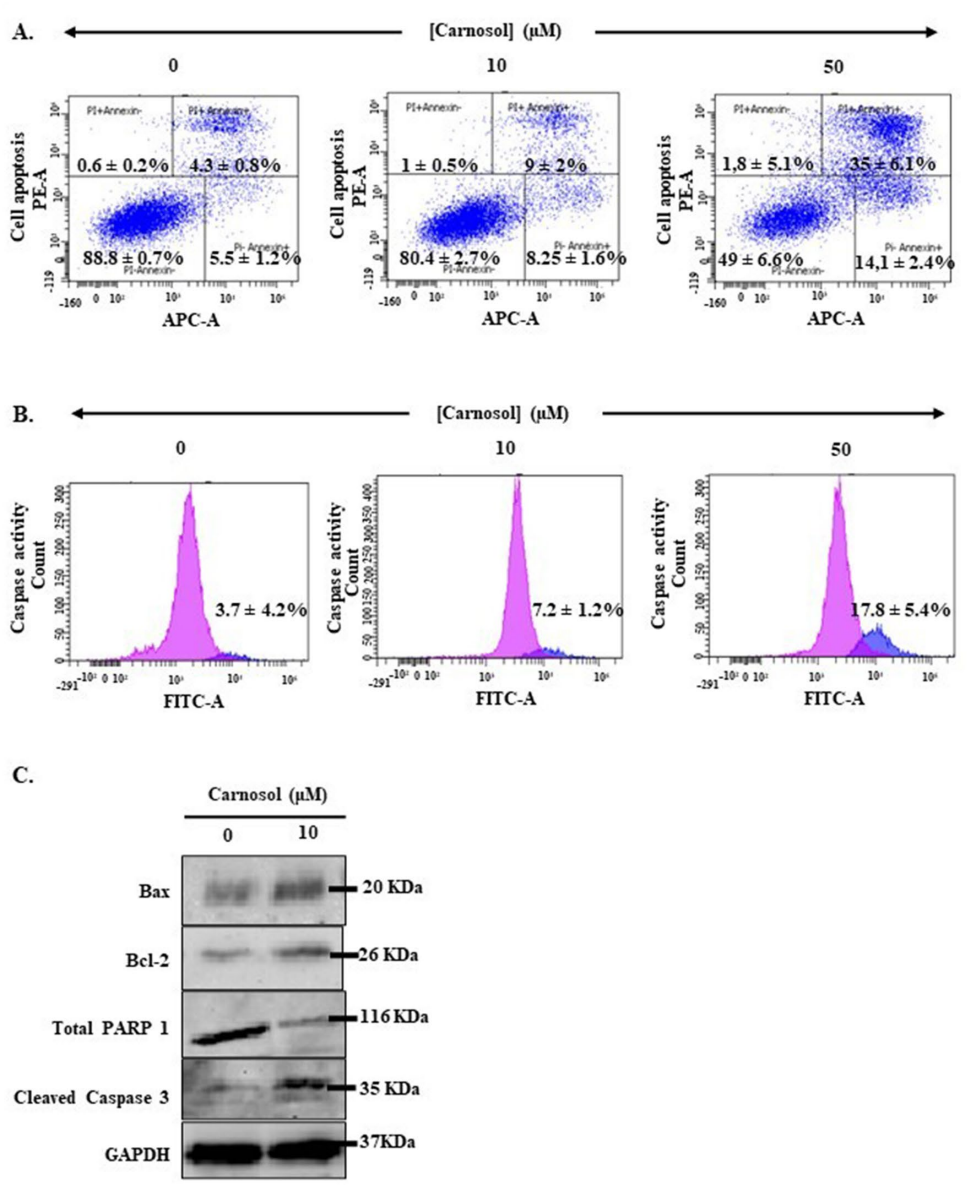
Effect of carnosol on Ca9-22 cell apoptosis. (**A**) Ca9-22 cell apoptosis after treatment with 10 and 50 µM carnosol. Cell populations are distinguished according to PI and Anx V expression levels. Dead cells were categorized into early apoptotic, late apoptotic, and necrotic (n = 4). (**B**) Caspase activity estimation (n = 3) and (**C**) Bax, Bcl2, total-PARP1, and cleaved caspase-3 protein expression levels after carnosol administration (n = 3).

The emergence of apoptotic cells highlight carnosol effectiveness. At 50 µM, the phytotherapy agent specifically stimulated early (14.1% ± 2.4%) and late apoptotic cells (35% ± 6.1%) as compared with the controls, where PI-/Annexin V + and PI + /Annexin+ population values measured 5.5% ± 1.2% and 4.3% ± 0.8%, respectively (Fig. 4A). This could be linked to the induction of active caspase formation and the modulation of multiple proapoptotic and antiapoptotic markers, including Bcl2, Bax, total-PARP1, and cleaved caspase-3. According to our findings, the percentage of Ca9-22 cells showing caspase activity increased in response to carnosol administration in a dose-dependent manner, from 3.7% ± 4.2% for control cells to 17.8% ± 5.4% for the 50 µM carnosol group (Fig. 4B). Moreover, western blots showed an increase in Bax expression level correlated with a decrease in Bcl-2 levels in the presence of carnosol. We also found that carnosol activated caspase-3 expression, which is a key pathway involved in apoptosis, thus confirming the results previously described in Fig. 4B. Therefore, cell death is likely activated upon stimulation of caspase signaling pathways (Fig. 4C).

**Figure 5:**
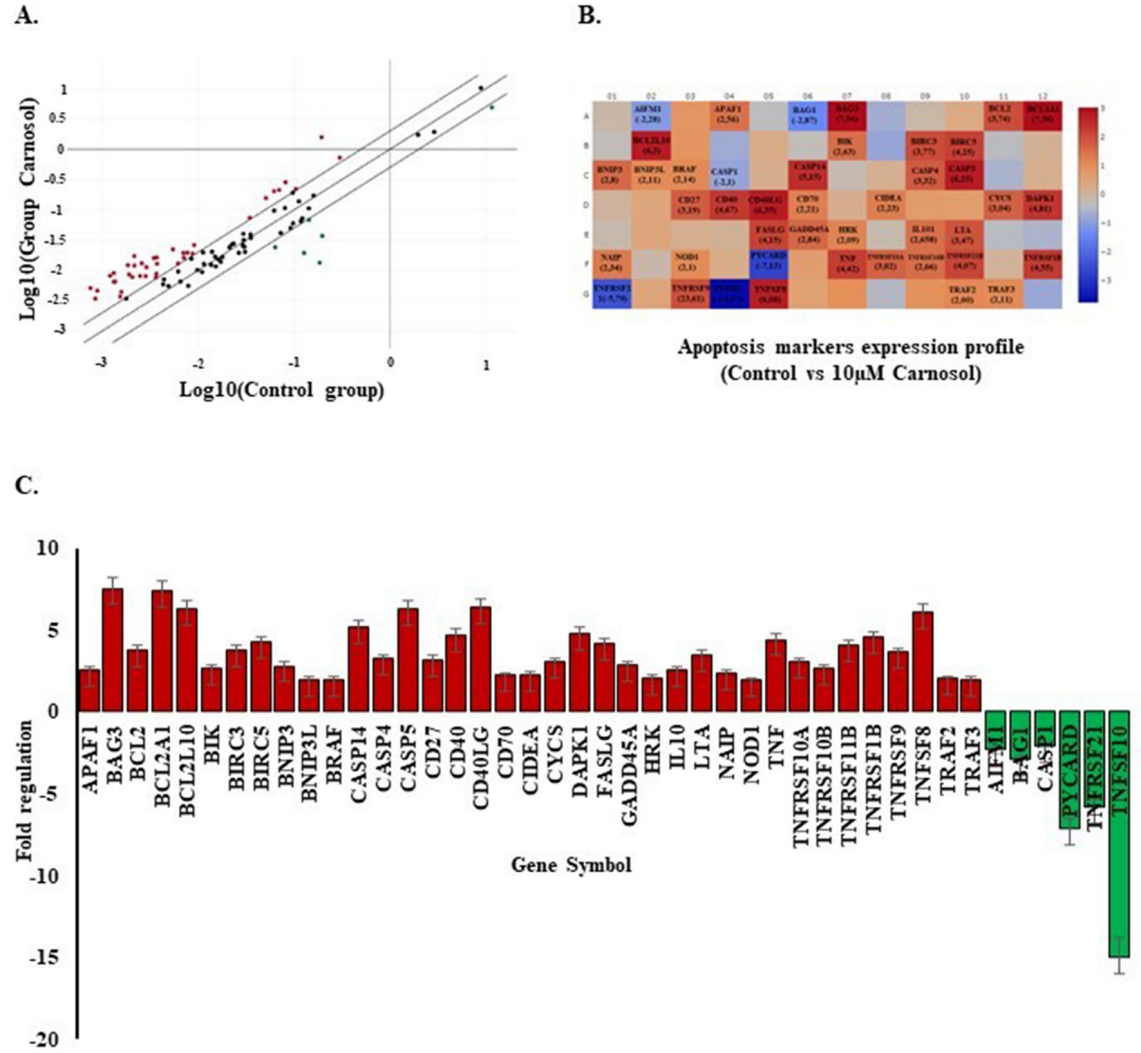
Effect of carnosol on Ca9-22 apoptotic marker expression profile. (**A**) QPCR array screening for 84 apoptotic markers after incubation with 10 µM carnosol. (**B**) and (**C**) Summary of positively and negatively modulated genes. Only fold regulation values > 2 were considered. The presented data are from two experiments.

Wide screening of 84 key apoptotic factors showed the modulation of 43 markers. Only fold regulation values > 2 were considered (Fig. 5A,B). Overall, 37 markers were upregulated, while only six, namely AIFM1 (Apoptosis Inducing Factor Mitochondria Associated 1), BAG1 (BCL2-associated athanogene 1), CASP1 (caspase 1), PYCARD, TNFRSF21, and TNFSF10, were downregulated (Fig. 5C).

### Carnosol controls Ca9-22 cell proliferation in a ROS-dependent manner

**Figure 6:**
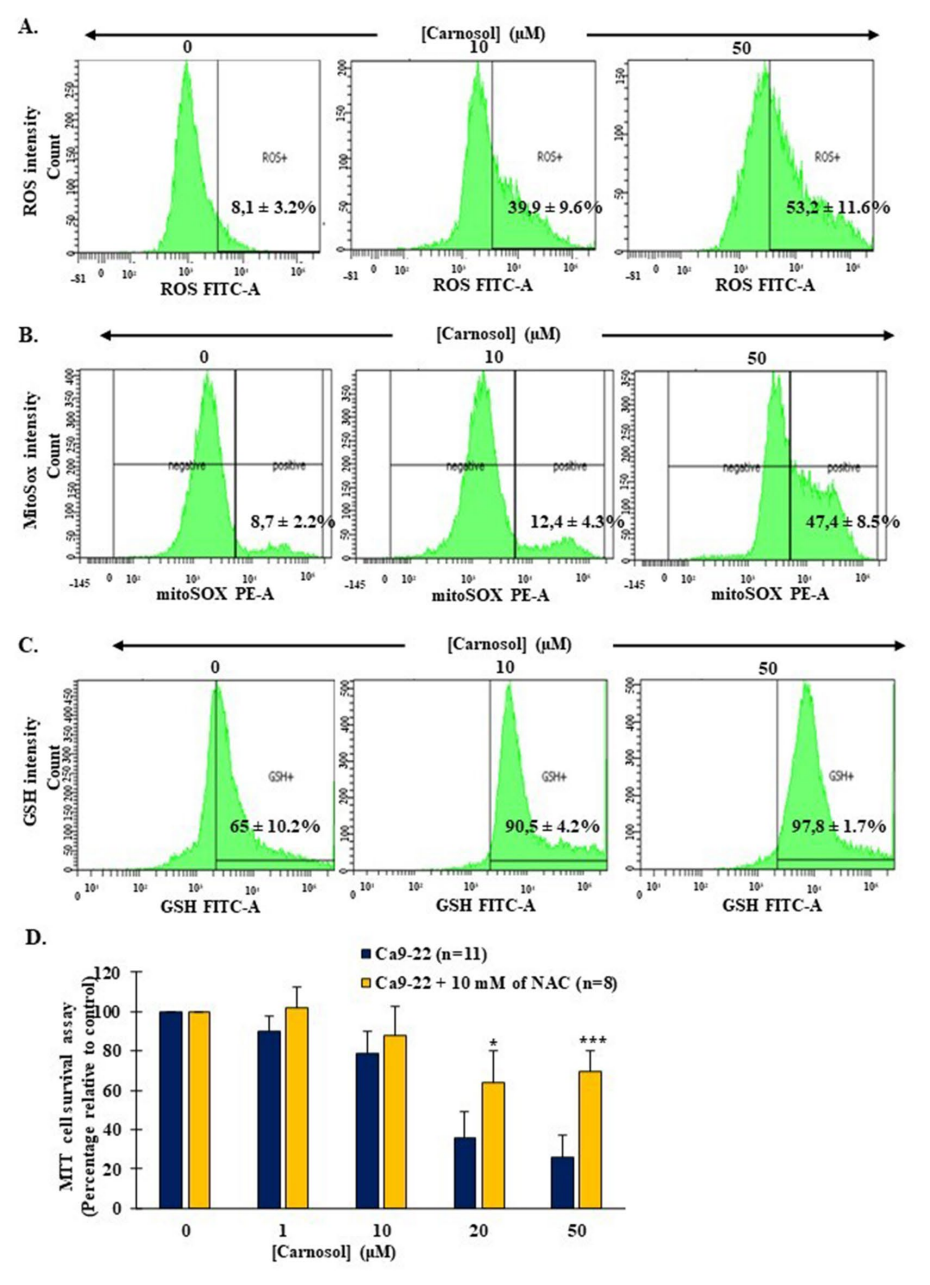
Effect of carnosol on Ca9-22 oxidative stress. (**A**) Total ROS expression levels assessed by flow cytometry (n = 4). (**B**) Mitochondrial ROS (n = 3) and (**C**) antioxidant GSH expression levels (n = 3) after treatment with 10 and 50 µM carnosol for 24 h. (**D**) MTT assay upon supplementation of different carnosol concentrations in the presence or absence of the ROS scavenger NAC. The presented data are mean ± SEM values from 8 to 11 independent experiments. *p < 0.05, **p < 0.01, ***p < 0.001, and ****p < 0.0001 are considered statistically significant.

Incubation of Ca9-22 cells with different carnosol concentrations for 24 h positively modulated total and mitochondrial ROS expression (Fig. 6A,B). Likewise, the antioxidant GSH expression level increased from 65% ± 10.2% to 90.5% ± 4.2% and 97.8% ± 1.7% by the actions of 10 and 50 µM carnosol, respectively (Fig. 6C). The addition of 10 mM ROS scavenger NAC counteracted the carnosol outcomes on proliferation. Significant results were observed in the 20 and 50 μM carnosol conditions. More specifically, when the cells were treated with 20 μM carnosol, cell viability was restored, and the values increased from 35.7% without NAC to 64.2% with NAC. With the highest carnosol concentration, 50 μM, the cell viability increased from 26% without NAC pretreatment to 69.7% with NAC pretreatment (Fig. 6D).

### Carnosol hinders Ca9-22 cell migration by blocking the epithelial-to-mesenchymal transition and extracellular matrix degradation

**Figure 7:**
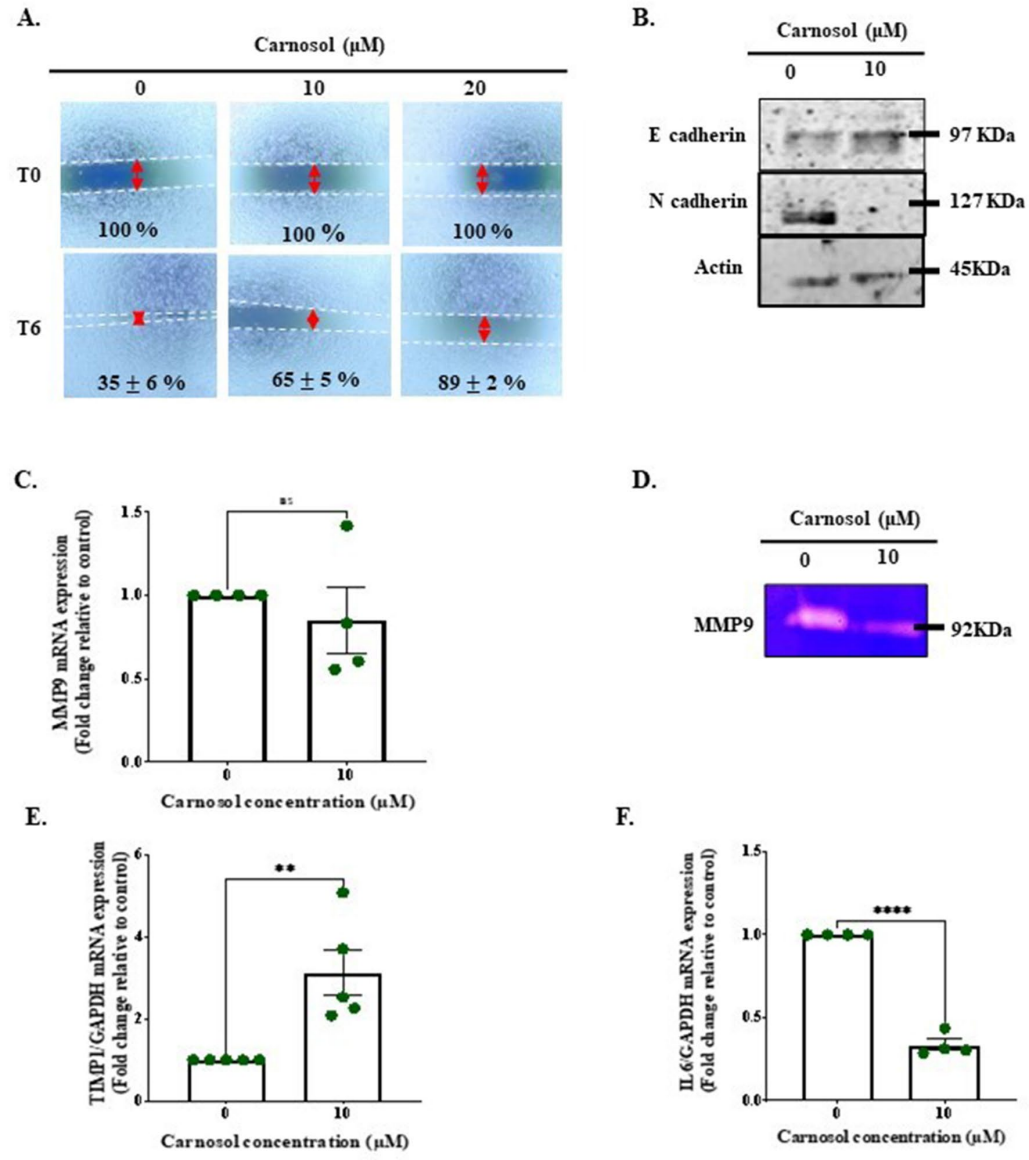
Effect of carnosol on Ca9-22 cell migration. (**A**) In the context of the scratch assay, wound diameter was measured at the beginning of the experiment and after 6 h of treatment with 10 and 20 μM carnosol (n = 6). (**B**) N- and E-cadherin adhesion molecules were investigated using western blotting analysis (n = 3). (**C**) and (**D**) This was correlated with intracellular and secreted MMP9 levels, as revealed by qPCR (n = 4) and gelatin zymography (n = 6). (**E**) TIMP1 and (**F**) IL6 mRNA levels measured using qPCR (n = 5 and n = 4, respectively). The presented data are mean ± SEM values from 3 to 6 independent experiments. *p < 0.05, **p < 0.01, ***p < 0.001, and ****p < 0.0001 are considered statistically significant.

Incubation with 10 and 20 μM carnosol for 6 h interfered with the scratch healing process, according to the scratch assay (Fig. 7A). A switch at the level of adhesion molecules, more specifically, from N cadherin to E cadherin, was also observed (Fig. 7B). Moreover, MMP9 secretion suppression (Fig. 7C,D) struck as the result of increased TIMP1 expression level (Fig. 7E). Lastly, this could be driven by the controlled management of the proinflammatory cytokine IL6 (Fig. 7F).

### Carnosol-mediated effects are controlled by multiple signaling pathways

**Figure 8:**
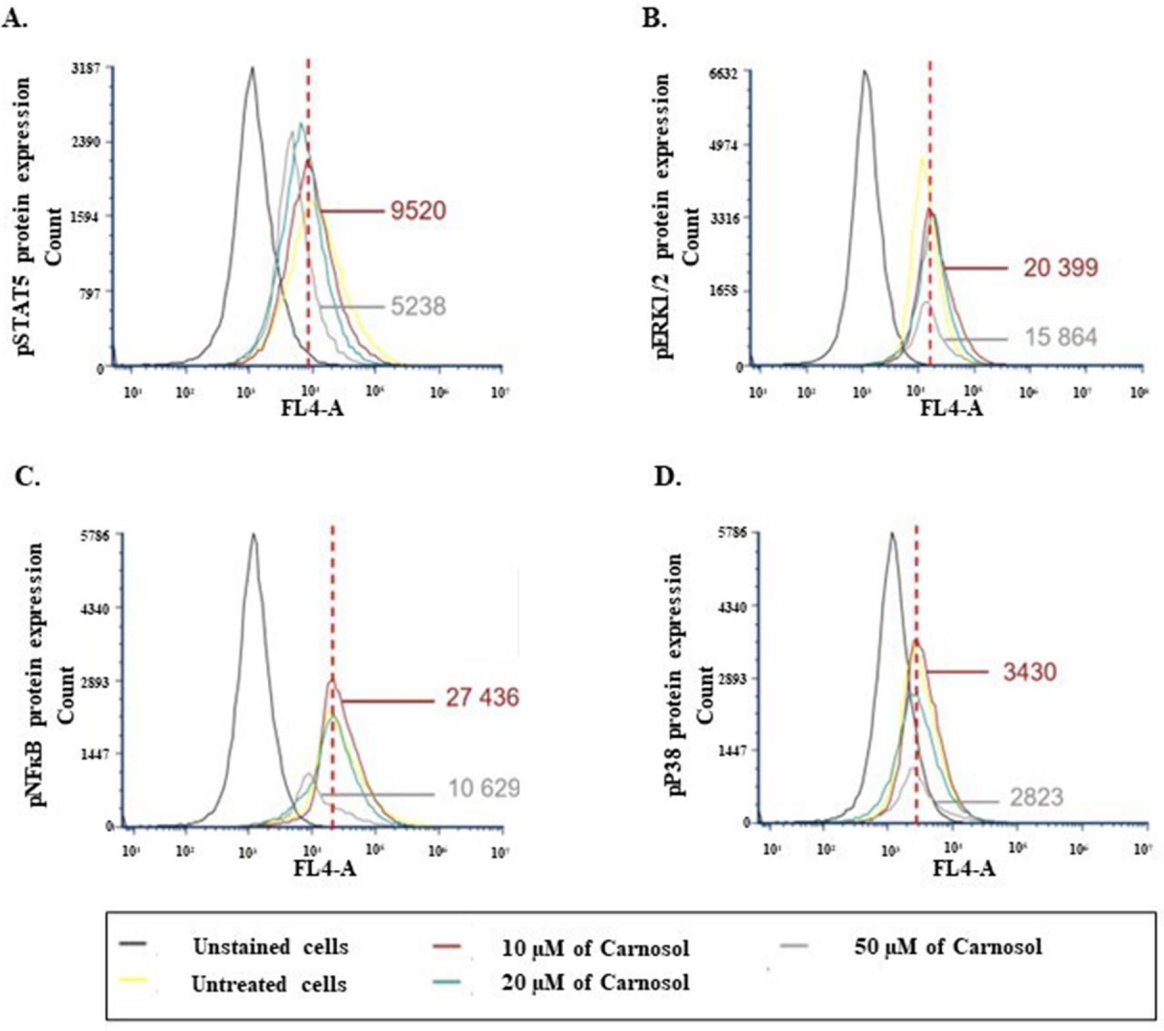
Effect of carnosol on the gingiva carcinoma signaling pathways. (**A**) STAT5, (**B**) ERK1/2, (**C**) NF-ĸB, and (**D**) P38 phosphorylation levels detected by flow cytometry after treatment of Ca9-22 cells with different carnosol concentrations for 24 h. Three independent experiments were conducted.

Flow cytometry results confirmed downregulation of the phosphorylated form of STAT5. The median fluorescence decreased from 9520 units in the untreated cells to 5238 units when the cells were stimulated by 50 μM carnosol (Fig. 8A). Carnosol also inhibited ERK1/ERK2 phosphorylation as the fluorescence values changed from 20,399 units to 515,864 units in the 50 µM carnosol group (Fig. 8B). Similar effects were observed regarding NF-ĸB activation and p38 phosphorylation. The fluorescence intensity decreased from 27,436 units in the control cells to 10,629 units and from 3430 units to 2823 units for pNF-KB and pp38, respectively (Fig. 8C,D). The most pronounced carnosol outcomes appears to be linked to the NF-ĸB and STAT5 transcription factors.

## Discussion

Gingiva carcinoma is considered an uncommon malignant neoplasm of the oral cavity. In fact, it constitutes approximately 10% of all diagnosed OCs^37^. Owing to the main affected areas, close to the teeth and periodontium, the tumor may adopt a benign inflammatory behavior. Early detection and effective treatment are crucial, as survival depends mainly on the stage of the disease. The aim of the present study was to examine the potential of carnosol as a complementary or alternative treatment for gingiva carcinoma by investigating its impact on cancer cell proliferation as well as on the underlying mechanisms involved. Our research findings show that low dose carnosol is tumor specific and causes a reduction in growth, colony formation, and cell viability by disrupting the cell cycle, particularly in gingiva carcinoma as compared with tongue cancer and normal epithelial cells. Carnosol-treated cells demonstrated alterations in many genes involved in cell cycle control, including cyclins (CCND2, CCNB2, CCNG1, and CCNG2), cyclin-dependent kinase (CDK2 and CDK5R1), and CDK-associated proteins (CKS2). Overall, the expression levels of tumor suppressor genes (BRAC-1 and p53) increased. These genes are known to suppress tumor growth and are commonly found to be inactive in many cancer types^38^ in opposition to quiescent cells^39^. In addition, their expression is governed by oncogenes such as c-myc.

Our qPCR array data showed that carnosol induced cell cycle arrest in OC mainly by upregulating the expression of genes involved in different phases, such as BCL2, BIRC5, BRCA1, CCND2, CDC6, CDK5R1, CKS2, E2F1, GADD45A, and MCM4. Nevertheless, Bcl-2 was widely reported to affect cell cycle progression through the management of the G0/G1 and G2/M phases. Bcl-2 overexpression has been associated with accelerated G1 arrest and delayed G1/S transition. Owing to growth factor deprivation, the cells presenting high BCL2 levels were smaller than the controls^40^. The lack of c-myc expression was also correlated with a reduction in nuclear size, which is considered an indicator of cell cycle arrest in G0 by BCL2-positive cells. This allows maintaining cell viability in abnormal conditions^40^. This is in line with BCL2 antiapoptotic function^41^. BIRC5 (Survivin) overexpression following carnosol administration can be explained by its ability to protect cells against various apoptotic stimuli such as exposure to chemotherapeutic drugs^42–44^. Survivin, described initially as a member of the anti-apoptotic protein family, also plays a key role as a regulator of the mitotic spindle checkpoint and a promotor of angiogenesis and chemoresistance^45^. Breast cancer susceptibility genes are tumor suppressor genes known to play a key role in DNA repair^46^, targeting G2/M cell cycle proteins^47^ and the maintenance of genomic stability^48^. BRCA1 overexpression by the effect of carnosol can be explained by the fact that it is closely linked to GADD45A and downstream signaling activation that lead to apoptotic cell death, as previously reported by Mullan et al. 2006^49^. CDKs play a crucial role in cell division and differentiation^50^. They were reported to control cancer cell migration, proliferation, apoptosis, and metastasis and therefore considered as potential therapeutic targets^50^. Our findings align with previous reports on the antiproliferation properties of carnosol in various tumor models^26,51^. Other natural drugs appear to decrease c-myc oncogene expression levels, triggering the activation of CDKIs and p53, thereby preventing cell cycle progression, especially at the G2/M stage^52,53^. Annurca apple polyphenol extract demonstrated a similar effect as carnosol on a triple-negative human breast carcinoma cell line (MDA-MB-23). It was found to selectively inhibit cell viability and to induce G2/M phase arrest^54^.

Our qPCR array data also disclose the downregulation of multiple other cell cycle markers, namely ATM, ATR, CCNB2, CCNG1, CCNG2, CDC20, CDK2, CDKN1B, CDKN2A, CDKN2B, CUL2, CUL3, MAD2L2, MDM2, RAD17, RB1, RBL2, STMN1, and WEE1. The members of the serine–threonine kinase family, ATM and ATR, are considered as key regulators of DNA damage. They were found to induce cell death through autophagy in response to oxidative stress^55^. Cyclin B2 (CCNB2), cyclin G1 (CCNG1), and cyclin G2 (CCNG2) are characterized by their capacity to bind CDKs and regulate their activities. Cyclin B2 was described as an oncogene that stimulates carcinogenesis both in vitro and in vivo^56^. Recent studies have identified cyclin G1 as a key component of the cyclin G1/Mdm2/p53 axis and as a strategic target for restoring cell cycle control through therapeutic gene transfer^57^. On the other hand, CDC20 was revealed as an oncoprotein that promotes human cancer progression^58^. The downregulation of CUL2 and CUL3 expression levels by carnosol is apparently linked to cyclin downregulation during the S-G2-M phases of the cell division cycle via glucose-dependent pathway^59^. Moreover, carnosol appeared to inhibit MAD2L2 expression, which is reported to be generally upregulated in tumors owing to aberrant mitotic activity. It is viewed as an indicator of chromosomal instability and correlated with reduced patient survival^60^. ATR/WEE1 kinases are highly expressed in various tumors, which makes them suitable targets for anticancer therapy. Their inhibition by carnosol can be an optimal approach for radio sensitization and chemosensitization^61^. The present study shows that carnosol significantly inhibits STMN1 expression. This oncoprotein is known for its ability to regulate cancer proliferation, cell cycle progression, mitotic division, and invasion, which are critical for tumorigenesis. It is also associated with taxane resistance such as in the paclitaxel case as well as with a poorer prognosis in various human cancers^62^.

Cell cycle arrest allows for either DNA damage repair or apoptosis induction. In severe cases, apoptosis is triggered after irreparable damage or failure to complete DNA repair during G2 arrest. Several studies have aimed to induce apoptosis as a therapeutic method for cancer treatment^63^. Our research has demonstrated that arrested Ca9-22 cells undergo apoptosis in response to carnosol. The underlying mechanism involves the activation of the mitochondrial pathway, highlighted by the upregulation of the Bax/Bcl-2 expression ratio. Bax triggers the release of cytochrome c from the mitochondria, which initiates caspase cascade activation and apoptosis induction. Carnosol stimulates many other proapoptotic genes such as the BCL2 family genes BCL2A1 and BCL2A10. Our qPCR array revealed that carnosol also modulated the expression levels of caspases and downregulated tumor necrosis factor (TNF) and TNF receptor (TNFR) superfamily genes. The dynamic changes in TNFR1-associated signaling complexes have been demonstrated to trigger the switch from inflammatory signaling to cell death via apoptosis or necroptosis^64^. These results are consistent with previous reports demonstrating that carnosol induces apoptosis in various cancer cell lines^65^. The balance between the proapoptotic protein Bax and the antiapoptotic protein Bcl-2 also permits the permeabilization of the mitochondrial outer membrane, which is a key event in the apoptosis intrinsic pathway. On a side note, the intrinsic pathway is initiated by internal cellular stress such as DNA damage or oxidative stress. Hence, in the Ca9-22 model, stress induction by carnosol can lead to the activation of the intrinsic pathway. This could be particularly useful in cases of cancer resistance to traditional chemotherapy drugs.

Apart from caspase-3, studies have shown that total PARP1 is considered a marker of apoptotic cell death. This enzyme is known to ensure DNA repair after cleavage by caspase-3. The expression levels of total PARP1 decreased in our study. One possible explanation is that carnosol treatment causes DNA damage, which activates the DNA repair function of PARP1, leading to its cleavage and depletion. Therefore, targeting PARP1 by carnosol administration could be a potential therapeutic strategy for cancer treatment. An increasing number of research reports have suggested that various types of polyphenols can regulate key steps involved in the apoptotic process. For example, genistein, an isoflavone that is a subclass of flavonoids found in various plant-based foods such as soybeans, has been shown to induce caspase-3 activation through cytochrome c release in bladder cancer cells^66^. Moreover, curcumin, a polyphenol found in turmeric, has been shown to induce Bcl-2 downregulation and Bax upregulation in OC cells^32,33^. A similar outcome was observed in a study conducted in 2014 where the authors provided strong evidence on apoptosis induction in triple negative breast cancer following carnosol administration. This encourages the recognition of this agent as an alternative therapeutic candidate for aggressive forms of breast tumors^67^.

Our research data also demonstrated that incubation of Ca9-22 cells with carnosol increased total and mitochondrial ROS as well as GSH levels. This indicates that the balance between ROS production and antioxidant defenses may be disrupted by carnosol treatment. Indeed, ROS may cause random damage to proteins, lipids, and DNA and may ultimately lead to cancer^68^. By inducing elevated GSH levels, carnosol enhances the antioxidant capacity of cells^69^. Increased mitochondrial superoxide levels can lead to the release of cytochrome c, a key factor in apoptosis induction^70^.

The final aim of the present study was to investigate the antimetastatic potential of carnosol by targeting tumor cell migration and invasion. Cancer cell metastatic activity is the basis for the aggressive behavior of tumors. Matrix metalloproteinases (MMPs) play a significant role in tumor invasion and metastasis by facilitating the breakdown of extracellular matrix components. Schieke et al. found that higher MMP levels are associated with tumorigenic characteristics^71^. Among the MMP family members, MMP-9 is particularly crucial because it is linked with tumor growth and metastasis in various malignancies^72^. Alterations that affect MMP levels can cause molecules such as cytochrome c to leak from the mitochondrial intermembrane space into the cytoplasm, which leads to apoptosis. In this study, we observed that downregulation of MMP-9 expression by carnosol administration was associated with tissue inhibitors of metalloproteinase 1 (TIMP1) upregulation and decreased expression level of interleukin 6 (IL-6), a pro-inflammatory cytokine. This is in accordance with other evidence that shows that MMP-9 expression is controlled by IL-6^73^. IL-6 has been shown to decrease TIMP-1 expression levels in OC cells^74^. Therefore, we suggest that carnosol originally decreases IL-6 levels, which leads to TIMP-1 upregulation and MMP-9 activity inhibition. Eugenol, a polyphenolic compound present in several plants such as clove, nutmeg, and cinnamon, was also found to inhibit the migration and invasion of different types of cancer cells by reducing the MMP expression levels^75^. This finding supports the antimetastatic activity of carnosol through a similar mechanism.

Finally, our study demonstrates that carnosol exhibits its effects on OC by decreasing the activation of key signaling pathways such as STAT5, ERK1/ERK12, p38, and NF-κB. JAK/STAT5 pathway induction has been linked to tumor growth and metastasis^76^. Abnormal STAT5 signaling in different types of cancers increases the expression levels of cyclin D, Bcl-2, and MMPs, among others, which ultimately leads to enhanced cell proliferation, survival, and metastasis^76^. The Raf/MEK/ERK cascade stimulates cell cycle arrest through the modulation of the p21 protein and therefore differentiation status. In cancer models, the activation of the ERK pathway enhances cell proliferation, survival, invasion, and metastasis^77^. The p38 pathway can also promote tumor cell migration, invasion, and metastasis by regulating the expression levels of multiple genes involved in these processes, such as c-myc^78,79^. The NF-κB pathway plays a crucial role in regulating immune response and cell survival. Aberrant activation of the NF-κB pathway in cancer can promote tumor growth, invasion, and metastasis. In addition, the activation of NF-κB in the mitochondria has been linked to the release of cytochrome c, which leads to caspase cascade initiation and, ultimately, programmed cell death^80^. The downregulation of the ERK, p38, and NF-κB pathways by carnosol administration is applicable in various types of cancer, which supports our findings on the anticancer effects of carnosol^81^.

## Conclusions

**Figure 9:**
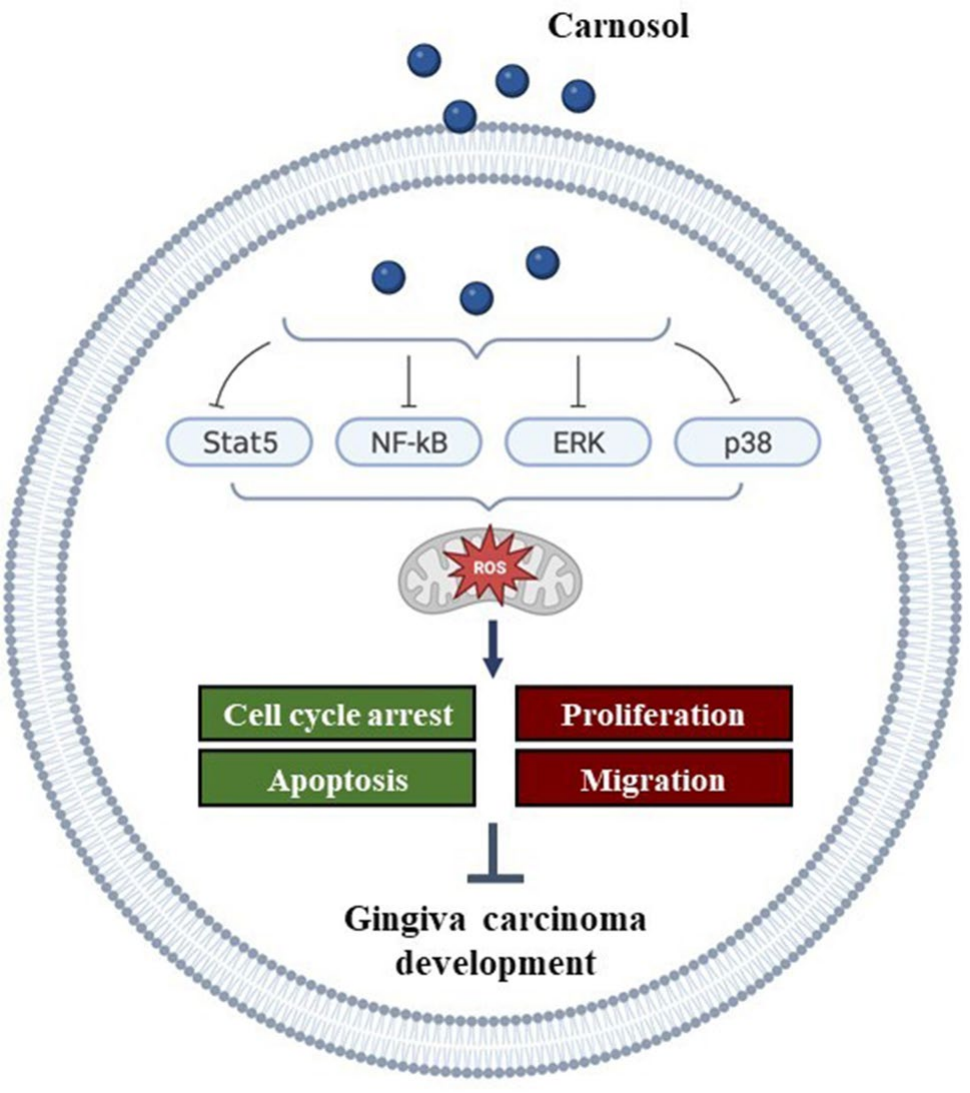
Schematic representation of carnosol’s mechanism of action in OC cells. Carnosol-mediated suppression of key cancer signaling pathways, namely pSTAT5, pERK1/2, pP38, and pNF-ĸB, is expected to cause DNA damage and subsequent initiation of ROS production. Oxidative stress controls cancer development through multiple mechanisms, including inhibition of gingiva carcinoma cell proliferation due to cell cycle arrest, obstruction of migration potential, and apoptosis induction.

To recap, the ability of Carnosol to inhibit cancer-promoting signaling pathways such as STAT5, NF-kB, ERK1/2 and p38 is believed to be responsible for its anti-gingiva carcinoma properties. This leads to oxidative stress and ROS generation that can be held accountable for cell proliferation, colony formation, MMPs activity and inflammation suppression. Apoptosis is also identified as a key player at this level. Carnosol’s general effects are summarized in Fig. 9.

## Data Availability

All data generated in the current study are included in this published article.
